# Ultrasound-Activated Metal–Organic Frameworks Incorporated Polyacrylonitrile Nanofibers Promote Macrophage Inflammation

**DOI:** 10.3390/nano16140853

**Published:** 2026-07-11

**Authors:** Shiqin Dai, Nao Kawata, Ahmed Nabil, Mitsuhiro Ebara

**Affiliations:** 1 Research Center for Macromolecules and Biomaterials, National Institute for Materials Science (NIMS), Tsukuba 305-0044, Ibaraki, Japan; s2336008@u.tsukuba.ac.jp (S.D.); s2630120@u.tsukuba.ac.jp (N.K.); tolba.ahmednabil@nims.go.jp (A.N.); 2 Graduate School of Pure and Applied Sciences, University of Tsukuba, Tsukuba 305-0006, Ibaraki, Japan; 3 Department of Materials Science and Technology, Tokyo University of Science, Tokyo 125-8585, Japan; 4 Department of Biochemistry and Microbiology, Faculty of Pharmaceutical Sciences, Chulalongkorn University, Bangkok 10330, Thailand

**Keywords:** piezoelectric nanofibers, metal–organic framework, macrophage polarization, electrical stimulation, immunomodulation

## Abstract

Macrophage polarization toward the pro-inflammatory M1 phenotype underlies an effective strategy for potentiating antitumor immune responses. Electrical stimulation has emerged as a potent modulator of immune cell polarization. However, conventional electrode-mediated electrical stimulation has limited penetration into deep tissues and relies on external power supplies. Here, we report on ultrasound (US)-responsive piezoelectric nanofibers, constructed by embedding manganese–titanium metal–organic frameworks (MT-MOF) within a polyacrylonitrile (PAN) matrix (MT-MOF/PAN). As a non-centrosymmetric bimetallic framework, MT-MOF generates a heterogeneous charge distribution under mechanical deformation, thereby enhancing the composite’s piezoelectric output. Furthermore, interfacial coupling between MT-MOF and the PAN nanofibers provides an additional contribution to this enhancement. Under US stimulation, MT-MOF/PAN nanofibers generates a peak voltage of 0.24 V, substantially exceeding the output of pure PAN nanofibers. In RAW-Blue cells, US-activated MT-MOF/PAN nanofibers significantly activate the nuclear factor-κB (NF-κB) pathway and promote the secretion of tumor necrosis factor-alpha (TNF-α) and interleukin-6 (IL-6), whereas neither US nor nanofibers alone produce this effect. Mechanistic studies demonstrate that piezoelectric stimulation induces a transient intracellular Ca^2+^ influx, as visualized by Fluo-4 acetoxymethyl ester (Fluo-4 AM) imaging, whereas US alone or nanofibers alone produce no significant effects. These findings establish MT-MOF/PAN nanofibers as a wireless, electrode-free platform for antitumor immunotherapy.

## 1. Introduction

Macrophage polarization is a fundamental determinant of innate immune outcomes, dictating phenotypic commitment toward either the pro-inflammatory M1 or the anti-inflammatory M2 state in a stimulus-contingent and microenvironment-dependent manner [1,2]. Sustained M1 polarization, marked by the overproduction of pro-inflammatory cytokines, such as tumor necrosis factor-alpha (TNF-α) and interleukin-6 (IL-6), are primary driver of tumor cytotoxicity. This polarization further remodels the immunosuppressive tumor microenvironment toward an immune-permissive state conducive to durable antitumor responses [3,4]. Precise control of macrophage phenotype therefore represents an attractive yet challenging therapeutic objective. Among emerging immunomodulatory modalities, electrical stimulation has attracted growing interest as a physical alternative to biological and pharmacological interventions. Owing to the inherent electrosensitivity of immune cells, this approach has demonstrated compelling efficacy in immune cell behavior [5,6]. Electrical cues directly influence plasma membrane potential, voltage-gated ion channel gating, and downstream intracellular signaling cascades. These include Ca^2+^-dependent and nuclear factor-κB (NF-κB) pathways, which are integral to pro-inflammatory cytokine gene expression [7,8]. Moreover, exogenous electric fields promote pro-inflammatory M1 polarization by activating guanine nucleotide exchange factor-H1 and its downstream NF-κB and AP-1/JNK pathways [9].

Despite this mechanistic promise, conventional electrical stimulation platforms rely on implanted or externally applied electrodes, which pose risks of infection and chronic tissue injury [10,11]. To circumvent these risks, piezoelectric materials provide a wireless, on-demand alternative, converting mechanical energy into localized electrical signals without an external power source or direct electrode contact [12,13]. Ultrasound (US) is a clinically established, non-invasive acoustic modality with deep tissue penetration capability, making it a promising remote stimulus for piezoelectric transducers [14]. Upon acoustic stimulation, piezoelectric surfaces undergo periodic deformation, inducing charge separation and generating transient surface electric fields that modulate adjacent cellular membranes [15]. Motivated by this capability, we sought to integrate piezoelectric functionality into our previously established implantable nanofiber platform.

In our previous studies, we developed a series of implantable, drug-loaded nanofibers with antitumor abilities. Oxaliplatin [16], lenvatinib [17], doxorubicin [18], paclitaxel [19], and temozolomide [20] were successfully incorporated into the nanofibers via an electrospinning method, enabling programmed drug release in response to external stimuli such as alternating magnetic fields [21,22]. To further extend this nanofibers-based strategy toward other functions, we herein focus on piezoelectric nanofibers. Polyacrylonitrile (PAN) is an electrospinnable polymer with intrinsic, albeit moderate, piezoelectric properties that arise from the dipole moments of its nitrile (–C≡N) groups [23,24]. Electrospinning under high electric fields induces partial alignment and polarization of these dipoles [25,26]. However, the piezoelectric output of neat PAN nanofibers remains insufficient for bioelectrical immunomodulation.

Metal–organic frameworks (MOF) have recently emerged as structurally tunable piezoelectric enhancers by virtue of their non-centrosymmetric crystal architectures, exceptionally high surface areas, and chemical designability [27]. The manganese–titanium metal–organic frameworks (MT-MOF) is particularly attractive in this context. Its heterogeneous charge distribution arises from the distinct electronegativity and coordination geometry of the metal nodes within the bimetallic framework, resulting in synergistic improvements in piezoelectric response under mechanical stress [28]. Moreover, the interfacial coupling between MOF and the PAN matrix has been shown to promote conversion from the 3_1_-helical to the piezoelectrically active planar zigzag conformation, thereby enhancing piezoelectric output [29]. Embedding MT-MOF within electrospun PAN nanofibers therefore represents a strategy to enhance composite piezoelectric output while preserving the flexibility for cell-material interactions.

**Scheme 1:**
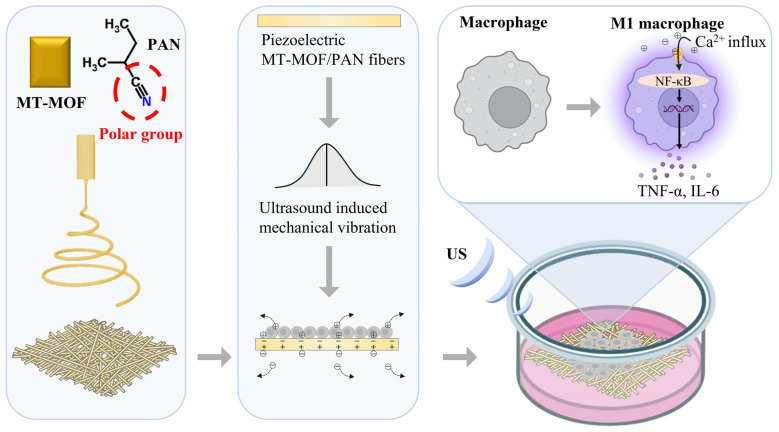
Schematic illustration of the fabrication of MT-MOF/PAN nanofibers via electrospinning and their application as US-responsive piezoelectric transducers for wireless, electrode-free macrophage M1 polarization in antitumor immunomodulation. Figure partially created in BioRender. Dai, S. (2026) https://BioRender.com/arr0xyh (accessed on 1 June 2026).

In the present study, we fabricated MT-MOF/PAN nanofibers by electrospinning and evaluated their structural, compositional, and piezoelectric properties (Scheme 1). We then examined the ability of US-activated MT-MOF/PAN nanofibers to promote M1 polarization in RAW-Blue cells, as evidenced by NF-κB activation and TNF-α/IL-6 secretion. Mechanistic investigations employing Fluo-4 acetoxymethyl ester (Fluo-4 AM) Ca^2+^ indicator further elucidated the cellular signaling basis of piezoelectric immunomodulation. This study represents the first demonstration of MT-MOF/PAN nanofibers that integrate the piezoelectric mechanism of bimetallic MOF with the electrospinnability of PAN for biomedical immunomodulation. This design provides a wireless, electrode-free strategy for macrophage M1 polarization that is distinct from PAN-only piezoelectric nanofibers. By establishing a mechanistic link between Ca^2+^ influx and NF-κB-mediated pro-inflammatory polarization, our findings suggest that MT-MOF/PAN nanofibers hold potential as a viable wireless electrical immunomodulatory platform for macrophage-based immunotherapy.

## 2. Materials and Methods

### 2.1. Materials and Reagents

The PAN powder (Mw, 1,500,000), Titanium (IV) isopropoxide (TTIP, ≥97% purity, stored under nitrogen) were purchased from Sigma-Aldrich Co. (St. Louis, MO, USA), while the 2-Aminoterephthalic acid (H_2_BDC-NH_2_, ≥98% purity) and Manganese (II) Chloride Tetrahydrate (MnCl_2_·4H_2_O, ≥98% purity) were obtained from Tokyo Chemical Industry Co., LTD. (Tokyo, Japan). N,N-Dimethylformamide (DMF, ≥99.5% purity) was provided by Fujifilm Wako Pure Chemical Co. (Tokyo, Japan). All above chemical reagents were stored at room temperature. Fetal bovine serum (FBS) was purchased from Tocris Bioscience Inc. (Minneapolis, MN, USA). High-glucose Dulbecco’s Modified Eagle Medium (DMEM), QUANTI-Blue, IL-6 enzyme-linked immunosorbent assay (ELISA) kit, TNF-α ELISA kit and AlamarBlue reagent were obtained from InvivoGen (San Diego, CA, USA). Hank’s Balanced Salt Solution (HBSS) was obtained from Thermo Fisher Scientific (Waltham, MA, USA). RAW-Blue cells (Cat. no. rawb-sp), derived from RAW 264.7 murine macrophages, were obtained from InvivoGen (San Diego, CA, USA). Dulbecco’s phosphate-buffered saline (PBS), paraformaldehyde, and Blocking One Histo were obtained from Nacalai Tesque (Kyoto, Japan). Fibronectin, 4′,6-Diamidino-2-phenylindole dihydrochloride (DAPI), and Triton X-100 were purchased from Sigma-Aldrich (St. Louis, MO, USA). Rhodamine phalloidin was obtained from Abcam (Cambridge, UK).

### 2.2. Synthesis of MT-MOF and MT-MOF/PAN Nanofibers

MT-MOF was synthesized according to a previously reported method [28]. A mixture of 54 mL DMF and 6 mL methanol was first prepared, and then 3.258 g H_2_BDC-NH_2_, 1.38 mL TTIP, and 0.0444 g MnCl_2_·4H_2_O were added to the mixture. The mixture was stirred for 20 min before being transferred into a Teflon-lined stainless-steel autoclave. The autoclave was heated to 150 °C for 36 h. Subsequently, the reactor was cooled to room temperature. The resulting MT-MOF was collected by centrifugation, washed three times each with DMF and methanol to remove unreacted raw materials, and dried under vacuum at 80 °C. MT-MOF was dispersed in 3 mL of DMF at a concentration corresponding to 10 wt.% relative to PAN, and the solution underwent 20 min of ultrasonic processing. Then, 10 wt.% PAN powder was added to the above solution and stirred for 2 h at 45 °C. An electrospinning machine Nanon-01A was used at a voltage of 25 kV (MECC Co., Ltd., Fukuoka, Japan). The resulting nanofibers was collected on an aluminum foil-covered plate at a spinning distance of 15 cm, using a blunt-tip stainless steel needle with an inner diameter of 0.4 mm and an outer diameter of 0.7 mm connected to a 5 mL syringe. Electrospinning was performed at room temperature and relative humidity (35 ± 10%). The flow rate was controlled at 0.3 mL h^−1^.

### 2.3. Characterization of MT-MOF and MT-MOF/PAN Nanofibers

The hydrodynamic diameter of MT-MOF was determined by dynamic light scattering (DLS, ELSZ-2000ZS, Otsuka Electronics, Osaka, Japan). The morphology of the MT-MOF and MT-MOF/PAN nanofibers was characterized by scanning electron microscopy (SEM, Hitachi SU8230, Hitachi, Tokyo, Japan), including low-angle secondary electron images (SE+SEL) and backscattered electron scanning electron microscopy images (BSE) of the two-dimensional planar membrane (BSE-TP). Image analysis was performed using ImageJ software (version 1.54p, National Institutes of Health, Bethesda, MD, USA) to quantify nanofibers diameters. Energy-dispersive X-ray spectroscopy (EDS) elemental mapping of SU8230 was used to characterize the distribution of elements. The accelerating voltage was set to 10 kV, and the working distance was adjusted to around 3 mm. A 3 nm platinum coating was applied before testing. Fourier transform infrared spectroscopy (FTIR) spectra were recorded with an FTIR spectrophotometer (Shimadzu IRAffinity-1S, Shimadzu, Kyoto, Japan). The crystallographic structures and composition were confirmed by X-ray diffraction (XRD, Rigaku MiniFlex, Co., Tokyo, Japan) with a scan range of 3–80° 2θ and a scan speed of 10° 2θ min^−1^ under 40 kV and 15 mA. An ultrasonic generator (KM-1200, Nanjing Guogong Ultrasonic Technology Co., Ltd., Nanjing, China) and 40 kHz US probe applied US to the nanofibers. To measure the direct voltage output under US stimulation, the nanofibers were sandwiched between two copper (Cu) electrodes (15 mm × 15 mm × 1 mm each) and encapsulated between top and bottom polyurethane (PU) insulating layers to minimize environmental interference. Subsequently, the two Cu layers were connected to the oscilloscope (DS1102 Z-E, Rigol Technologies, Inc., Beijing, China) through a voltage probe (PVP3150, Rigol Technologies, Inc., Beijing, China) with a 10-megohm input impedance to record the output voltage. US was applied at 1200 W power and 40 kHz frequency for 1.2 s per measurement. Testing was performed at room temperature in ambient air.

### 2.4. Cell Culture

RAW-Blue cells were cultured in high-glucose DMEM supplemented with 10% FBS and 1% penicillin-streptomycin at 37 °C in a humidified atmosphere containing 5% CO_2_ (MCO-170AICUVH, PHC Holdings Corporation, Tokyo, Japan). The used cell passage was between passages 4 and 7. Electrospun nanofibers were cut into squares (25 mm^2^), washed with 77% ethanol and PBS. Before starting the cell culture, the nanofibers were placed on the 96-well plate and sterilized by ultraviolet (UV) irradiation for 5 min under a cabinet UV crosslinker emitting 254 nm UV light (UVP Crosslinker CL-3000, Analytik Jena, Upland, CA, USA). The nanofibers were then coated with 50 μL of fibronectin (20 μg mL^−1^) in a 96-well plate for 2 h at 37 °C, followed by one PBS wash before cell seeding. RAW-Blue cells were seeded at a density of 200,000 cells per well on the sterilized nanofibers, and cultured in high-glucose DMEM for 2 h to allow cell attachment. Subsequently, the cell-seeded nanofibers were gently rinsed with PBS, transferred to 24-well plates, and cultured for an additional 24 h. US stimulation was then applied for 40 min. Finally, the culture supernatants were collected for subsequent analysis.

### 2.5. Cell Viability Assessment and Hemocompatibility Assessment

Cell viability was quantified using the AlamarBlue assay. Following treatment, cells were incubated with AlamarBlue reagents diluted in culture media (10%) for 4 h at 37 °C. Medium was then sampled and analyzed using a fluorescence plate reader (Infinite M Nano+, Tecan Japan, Kawasaki, Japan). Viability was expressed as a percentage of the fluorescence intensity in untreated control cells. Hemocompatibility was evaluated by a hemolysis assay. Fresh porcine whole blood (Tokyo Shibaura Zouki Co., Ltd., Tokyo, Japan) was centrifuged at 3000 rpm for 3 min to collect red blood cells (RBCs), which were washed with PBS and diluted to a 10% (*v*/*v*) suspension. PAN and MT-MOF/PAN nanofiber samples were incubated with the RBCs suspension for 1 h under gentle rotation, using PBS and 0.2% (*v*/*v*) Triton X-100 as negative and positive controls with a final ratio of 2% (*v*/*v*) RBCs, respectively. After incubation, the mixtures were centrifuged at 10,000 rpm for 2 min, and the absorbance of the supernatant was measured at 540 nm. The hemolysis ratio was calculated as follows: Hemolysis ratio (%) = (A_sample_ − A_negative_)/(A_positive_ − A_negative_) × 100%.

### 2.6. Cell Staining

Intracellular reactive oxygen species (ROS) were measured using an ROS assay kit (Cat. No. R253, Dojindo Laboratories, Kumamoto, Japan) according to the manufacturer’s instructions and imaged with a fluorescence microscope (Eclipse Ti2-E microscope, Nikon Corporation, Tokyo, Japan). After the AlamarBlue assay, cells were fixed with 4% (*w*/*v*) paraformaldehyde for 15 min and permeabilized with 0.2% (*v*/*v*) Triton X-100 for 2 min. After blocking with Blocking One Histo for 20 min, F-actin was stained with rhodamine phalloidin for 30 min. Cell nuclei were counterstained with DAPI for 2 min. Fluorescence images were acquired using a fluorescence microscope and analyzed with ImageJ software.

### 2.7. Cytokines Quantification

Culture supernatants were collected after stimulation. The activation of the NF-κB pathway was quantified by QUANTI-Blue kit, with substrate incubation time and temperature standardized across all samples to minimize variability from the time-dependent colorimetric reaction. Absorbance was read at 450 nm with a reference wavelength of 570 nm. TNF-α and IL-6 were quantified by TNF-α ELISA kit and IL-6 ELISA kit following the manufacturer’s protocols, with a standard curve included on every plate to control for plate-to-plate variation.

### 2.8. Intracellular Ca^2+^ Imaging by Fluo-4 Acetoxymethyl Ester

Intracellular Ca^2+^ dynamics were monitored using the membrane-permeant indicator Fluo-4 AM (Abcam, Cambridge, UK). Cells were loaded with 5 μM Fluo-4 AM in HBSS containing 0.04% (*w*/*v*) Pluronic F-127 for 10 min at 37 °C. Ca^2+^ transients were acquired after US stimulation by fluorescence microscope at an excitation wavelength of 495 nm and emission wavelength of 528 nm. Peak fluorescence responses were expressed as ΔF/F_0_, where ΔF is the change in fluorescence intensity from baseline (F_0_).

### 2.9. Statistical Analysis

All quantitative data are presented as mean ± standard deviation (SD) from a minimum of three independent experiments. Statistical comparisons across multiple groups were performed by Student’s *t*-test and one-way analysis of variance (ANOVA) followed by Tukey’s honestly significant difference (HSD) post hoc test. * *p* < 0.05, ** *p* < 0.01; n.s., not significant. All analyses were performed using SPSS Statistics 19 (Version 19, IBM Corp., Armonk, NY, USA).

## 3. Results and Discussion

### 3.1. Characterization of MT-MOF and MT-MOF/PAN Nanofibers

To characterize the morphology and particle size of the synthesized MT-MOF, SEM and DLS measurements were carried out, and the results were presented in Figure S1. SEM imaging revealed that MT-MOF possesses a cubic morphology (Figure S1a). The corresponding particle size distribution obtained from DLS showed a predominant peak at approximately 351 nm (Figure S1b), accounting for the majority of the particle population.

**Figure 1:**
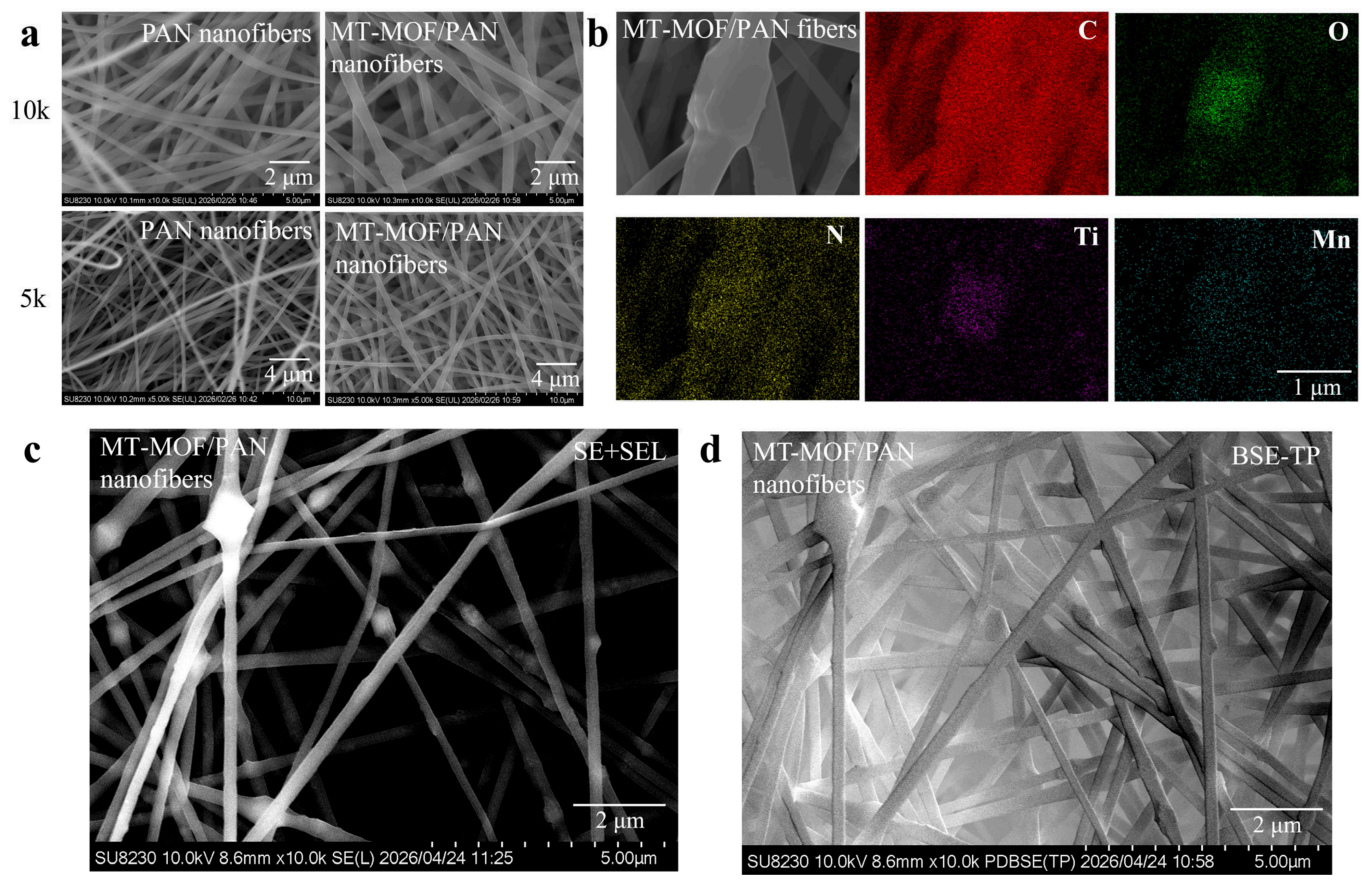
(**a**) SEM of PAN nanofibers and MT-MOF/PAN nanofibers, and (**b**) EDS, (**c**) SE+SEL, and (**d**) BSE-TP of MT-MOF/PAN nanofibers.

The morphology and elemental distribution of MT-MOF/PAN composite nanofibers were characterized to confirm the successful fabrication of the nanofibers platform. As shown in Figure 1a, no significant bead formation or nanofibers fusion was observed, reflecting the appropriate viscosity and conductivity of the spinning solution. Notably, MT-MOF nanoparticles were observed exclusively in MT-MOF/PAN nanofibers. ImageJ analysis yielded average nanofibers diameters of 329 nm for PAN nanofibers and 521 nm for MT-MOF/PAN nanofibers, with the latter showing a broader size distribution (Figure S2a,b). The increased nanofibers diameter of MT-MOF/PAN nanofibers relative to PAN nanofibers was attributed to the successful loading of MT-MOF into the PAN nanofibers. To further verify the successful incorporation of the MT-MOF within the nanofibers, EDS mapping was conducted. As presented in Figure 1b, the elemental mapping showed the distribution of Mn, Ti, O, and C elements throughout the fibrous network, with pronounced Ti and Mn signals localized within the nanoparticle regions, confirming the successful incorporation of MT-MOF. Quantitative EDS analysis further revealed that the sample was composed of C (78.13 wt%), N (14.23 wt%), along with O (4.76 wt%), Ti (2.61 wt%), and Mn (0.27 wt%). Diluted within the carbon-rich MT-MOF/PAN nanofibers, Ti and Mn were present at low contents, resulting in relatively weak EDS signals. The Mn signal was less pronounced than Ti, reflecting its role as a minor dopant rather than an equimolar bimetallic constituent. This asymmetric incorporation is thought to give rise to Mn-Ti-oxo units with heterogeneous charge distribution, potentially contributing to the piezoelectric properties of the nanoparticles. To further visualize the MT-MOF distribution, SE+SEL and BSE-TP images were acquired (Figure 1c,d). The enhanced atomic-number contrast in SE+SEL clearly distinguished the MT-MOF particles from the polymer matrix, while BSE-TP further revealed their three-dimensional distribution within the fibers, together confirming the uniform embedding of MT-MOF throughout the nanofibers structure.

**Figure 2:**
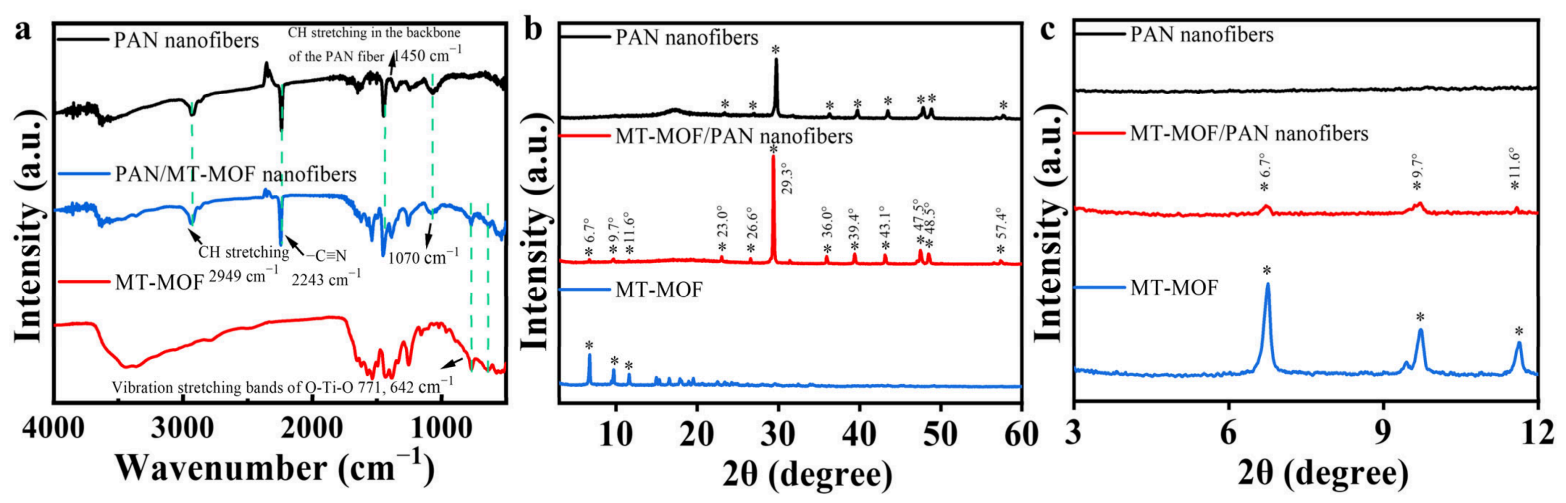
(**a**) FTIR, (**b**) XRD patterns over the full scanning range (2θ = 3–80°) of MT-MOF, PAN nanofibers, MT-MOF/PAN nanofibers, (**c**) Enlarged XRD patterns in the low-angle region (2θ = 3–12°) of MT-MOF, PAN nanofibers, MT-MOF/PAN nanofibers. Asterisks (*) indicate the characteristic peaks.

FTIR spectroscopy was used to identify the characteristic chemical bonds of MT-MOF, PAN nanofibers, and MT-MOF/PAN nanofibers. As shown in Figure 2a, the PAN nanofibers exhibited characteristic absorption bands at 2243 cm^−1^ corresponding to the C≡N stretching vibration, and bands near 1450 cm^−1^ attributed to CH stretching in the backbone of the PAN nanofibers [30]. For MT-MOF, stretching vibration bands of O–Ti–O were observed at 771 cm^−1^ and 642 cm^−1^ [31]. Additionally, asymmetric and symmetric carboxylate vibrations appeared at 1435/1539 cm^−1^ and 1388/1575 cm^−1^, respectively, confirming the formation of the MT-MOF. The spectrum of MT-MOF/PAN nanofibers retained characteristic peaks from both MT-MOF and PAN, confirming the coexistence of both components. The crystallinity and phase composition of MT-MOF, PAN nanofibers, and MT-MOF/PAN nanofibers were analyzed using XRD (Figure 2b). The XRD pattern of pure PAN nanofibers showed a broad diffraction peak centered around 23.0°, 26.6°, 29.3°, 36.0°, 39.4°, 43.1°, 47.5°, 48.5°, and 57.4°, indicative of its semi-crystalline polymeric nature. In contrast, the MT-MOF exhibited multiple sharp diffraction peaks corresponding to its crystalline framework. The XRD pattern of MT-MOF/PAN nanofibers showed characteristic peaks from MT-MOF at around 6.7°, 9.7°, and 11.6° in enlarged XRD patterns, indicating that the crystal structure of MT-MOF was preserved during the composite fabrication process, while there was no characteristic peak of MT-MOF in the XRD pattern of PAN fibers (Figure 2c). The relatively low intensity of MT-MOF peaks in the composite was consistent with the 10 wt.% MOF loading diluted within the PAN matrix. PAN crystallinity was not significantly altered by MT-MOF incorporation, as evidenced by the unchanged position and breadth of the PAN characteristic peaks. Taken together, the multi-technique approach of SEM, EDS, FTIR, XRD, and BSE-TP imaging collectively provided evidence for the successful incorporation of MT-MOF into PAN nanofibers.

### 3.2. Characterization of Piezoelectric Properties

**Figure 3:**
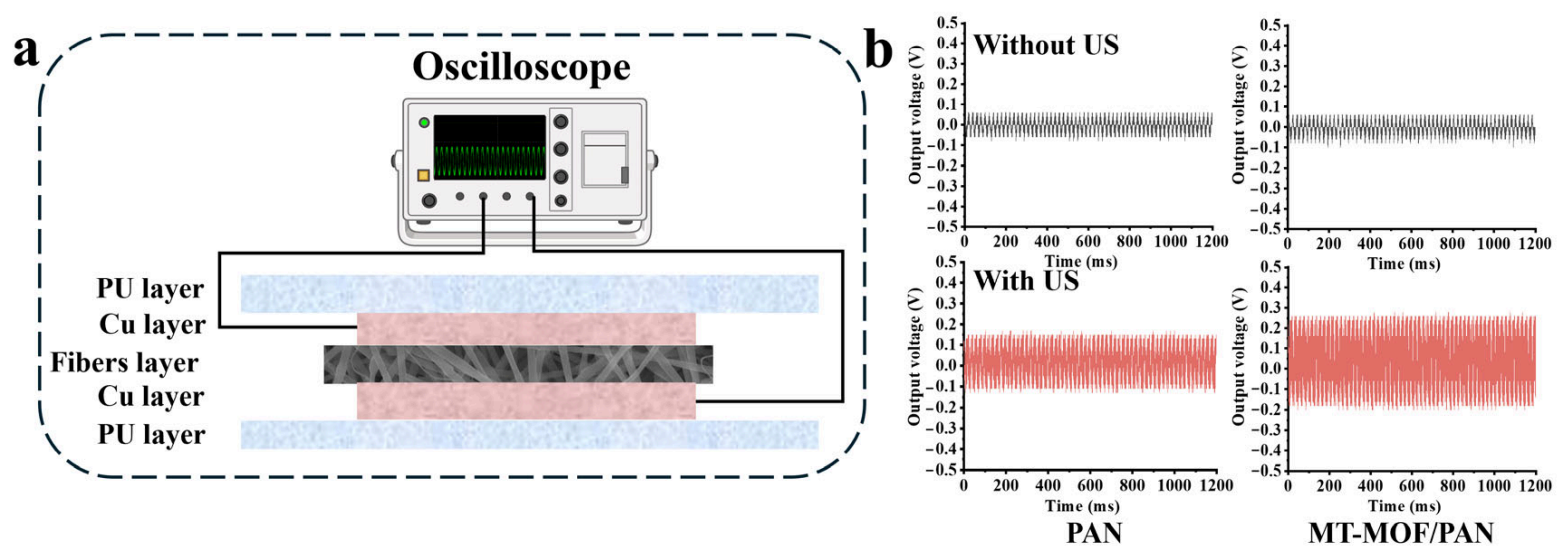
Characterization of piezoelectric properties. (**a**) Schematic diagram of the sandwich structure for measuring output voltage of nanofibers, (**b**) Output voltage of PAN nanofibers, MT-MOF/PAN nanofibers under the US stimulation.

To evaluate the piezoelectric behavior of the MT-MOF/PAN nanofibers under US stimulation, a sandwich-structured device was assembled for output voltage measurement (Figure 3a). The nanofibers were exposed to US excitation. The mech anical oscillation induced by the acoustic wave generates periodic stress on the piezoelectric nanofibers, which in turn produces alternating electric fields due to deformation-induced dipole reorientation within the nanofibers. The PAN nanofibers exhibited a relatively low piezoelectric output, generating a voltage of 0.12 V (Figure 3b). This is attributed to the inherently weak piezoelectricity of PAN, which possesses polar nitrile groups but limited crystallinity and dipole moments in nanofibers. The intrinsic piezoelectric properties of MT-MOF have been previously characterized [28] and originate from the mechanoelectrical transduction of its non-centrosymmetric bimetallic crystal architecture. This structure generates a heterogeneous charge distribution and contributes to the net piezoelectric response under stress. Furthermore, the interfacial coupling between MOF and the PAN matrix has been shown to promote conversion from the 3_1_-helical to the piezoelectrically active planar zigzag conformation, thereby enhancing piezoelectric output [29]. In contrast, the composite nanofibers loaded with MT-MOF showed an increase in output voltage. The 10 wt.% MT-MOF/PAN nanofibers delivered a peak voltage of 0.24 V. These results indicated that the incorporation of MT-MOF into PAN nanofibers enhanced the generation of electric fields under US stimulation.

### 3.3. Biosafety Evaluation

**Figure 4:**
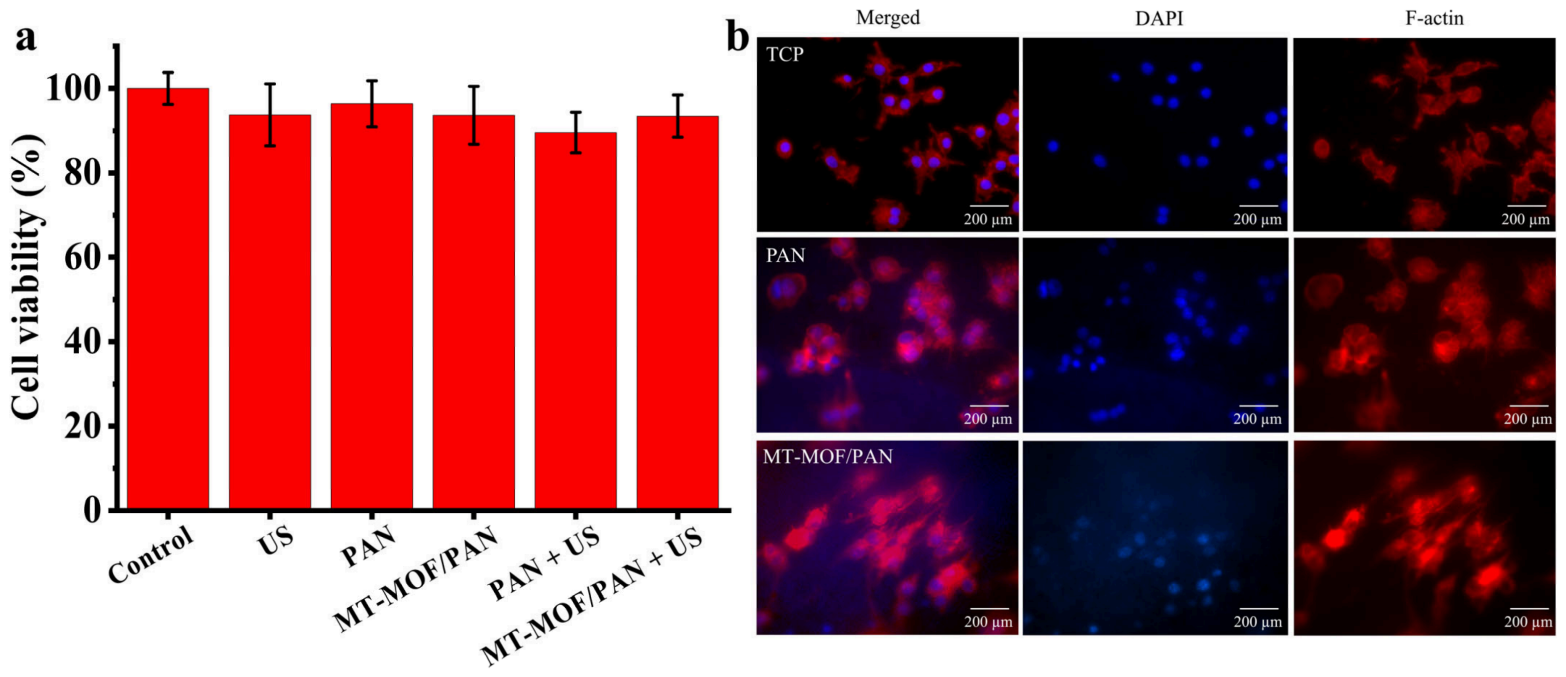
(**a**) Cell viability of control, US, PAN, MT-MOF/PAN, PAN + US, and MT-MOF/PAN + US groups, (**b**) Immunofluorescence images of cells on TCP group, PAN group, and MT-MOF/PAN group (n = 3). Individual channels for DAPI (nuclei) and F-actin (cytoskeleton) with the corresponding merged pseudo-colored image (DAPI: blue, F-actin: red).

To investigate the biosafety of PAN nanofibers, MT-MOF nanofibers and applied US, the AlamarBlue assay and F-actin/DAPI staining were used. RAW-Blue cell viability exceeded 90% across all experimental groups, confirming the cytocompatibility of both the nanofibers and the applied US protocol (Figure 4a). The adhesive structure of RAW-Blue cells can be altered by different substrates. Therefore, we stained cells with F-actin and DAPI to observe the adherent cells on tissue culture polystyrene (TCP), PAN, and MT-MOF/PAN at 24 h (Figure 4b). Low-magnification overview images confirmed cell distribution and substrate adhesion across groups. Cells exhibited significantly larger spread areas on the PAN nanofibers and MT-MOF/PAN nanofibers than on TCP, with no significant difference between the two nanofiber groups (Figure S3a). Solidity was defined as the ratio of the nucleus to its convex hull [32]. It showed no significant difference across three groups, indicating that the cells retained smooth, regular contours without the protrusive or blebbing irregularities [33] (Figure S3b). Combined with the enlarged spread area and the high viability in the AlamarBlue assay, these results indicated that the PAN and MT-MOF/PAN nanofibers’ topographies supported cell spreading while remaining cytocompatible, making them suitable substrates for cell culture and macrophage regulation.

To assess whether the nanofibers induce oxidative stress, intracellular ROS staining was performed, with H_2_O_2_ treatment included as a positive control to validate the assay and to establish the appearance of genuine ROS-positive cells against the probe-adsorption background of the nanofibers. ROS staining showed minimal fluorescence in untreated control cells and markedly enhanced fluorescence after H_2_O_2_ treatment, confirming successful induction of oxidative stress. The +H_2_O_2_ +PAN group, as a positive control, showed numerous distinct fluorescent cells, whereas the +PAN and +MT-MOF/PAN groups, regardless of US treatment, displayed negligible fluorescent cell signals, confirming the good biocompatibility of the PAN and MT-MOF/PAN scaffolds under both static and US-stimulated conditions (Figure S4). In addition, a hemolysis assay was performed to evaluate the hemocompatibility of the nanofibers. As shown in Figure S5a, the PBS group remained clear, while the Triton X-100 and water groups showed an obvious red color due to complete hemolysis. Both the PAN and MT-MOF/PAN groups exhibited results comparable to PBS, with intact RBCs after centrifugation. The quantitative hemolysis ratios of PAN nanofibers and MT-MOF/PAN nanofibers were 0.37% and 0.84%, respectively. Both values were well below the 2% safety threshold, confirming their hemocompatibility (Figure S5b). Collectively, these results demonstrated that both the PAN nanofibers and MT-MOF/PAN nanofibers are cytocompatible and hemocompatible, supporting their suitability for subsequent cellular applications.

### 3.4. Cytokine Quantification

**Figure 5:**
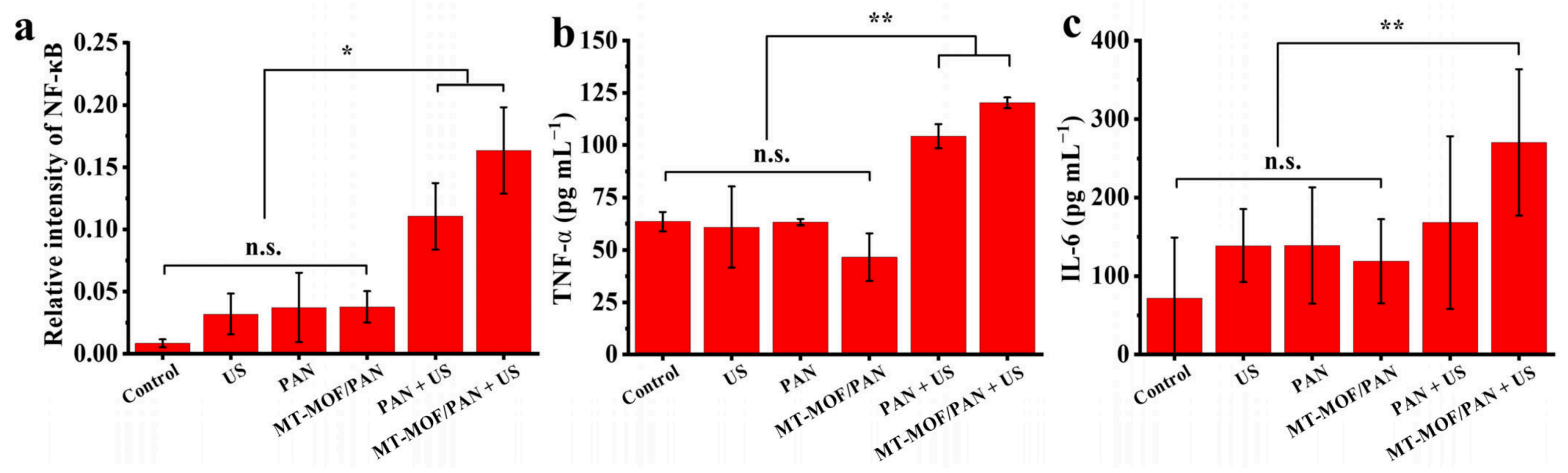
(**a**) NF-κB expression, (**b**) TNF-α secretion, and (**c**) IL-6 secretion of control, US, PAN, MT-MOF/PAN, PAN + US, MT-MOF/PAN + US groups. (n = 3), * *p* < 0.05, ** *p* < 0.01, n.s. for not significant.

To investigate the effect of PAN nanofibers and MT-MOF/PAN nanofibers on macrophage polarization, US was applied to cultured cells for noninvasive and noncontact stimulation. Activation of the NF-κB pathway promotes the differentiation of macrophages into the M1 phenotype [34]. TNF-α and IL-6 are typical markers of M1 macrophages [35]. Activation of the NF-κB pathway was quantified by QUANTI-Blue kit, and the secretion of TNF-α and IL-6 was quantified by ELISA kit across all experimental groups. NF-κB expression was highest in the MT-MOF/PAN + US group, reaching a relative intensity of 0.16, significantly higher than that in the control group at a relative intensity of 0.01. Intermediate levels were observed in the PAN + US group at a relative intensity of 0.11. At the same time, there was no significant difference among the control, US, PAN, and MT-MOF/PAN groups, indicating that US alone and nanofibers alone did not produce significant effects (Figure 5a). Similarly, TNF-α secretion was increased in the PAN + US group at 104 pg mL^−1^ and the MT-MOF/PAN + US group at 120 pg mL^−1^, which were significantly higher than the control at 64 pg mL^−1^. Also, no significant differences were observed among the control, US, PAN, and MT-MOF/PAN groups, indicating that TNF-α secretion was not influenced by US alone or nanofibers alone, and that neither US nor the nanofiber substrate independently drives TNF-α upregulation (Figure 5b). In contrast, the combination of nanofibers and US significantly elevated TNF-α, confirming that the concurrent presence of the nanofibers and US stimulation was required for the pro-inflammatory cytokine response. IL-6 secretion showed a similar trend, peaking in the MT-MOF/PAN + US group at 270 pg mL^−1^, significantly higher than the control at 72 pg mL^−1^ (Figure 5c). Collectively, these results demonstrated that the nanofibers platform + US potentiated pro-inflammatory cytokine secretion.

### 3.5. Mechanistic Investigation

**Figure 6:**
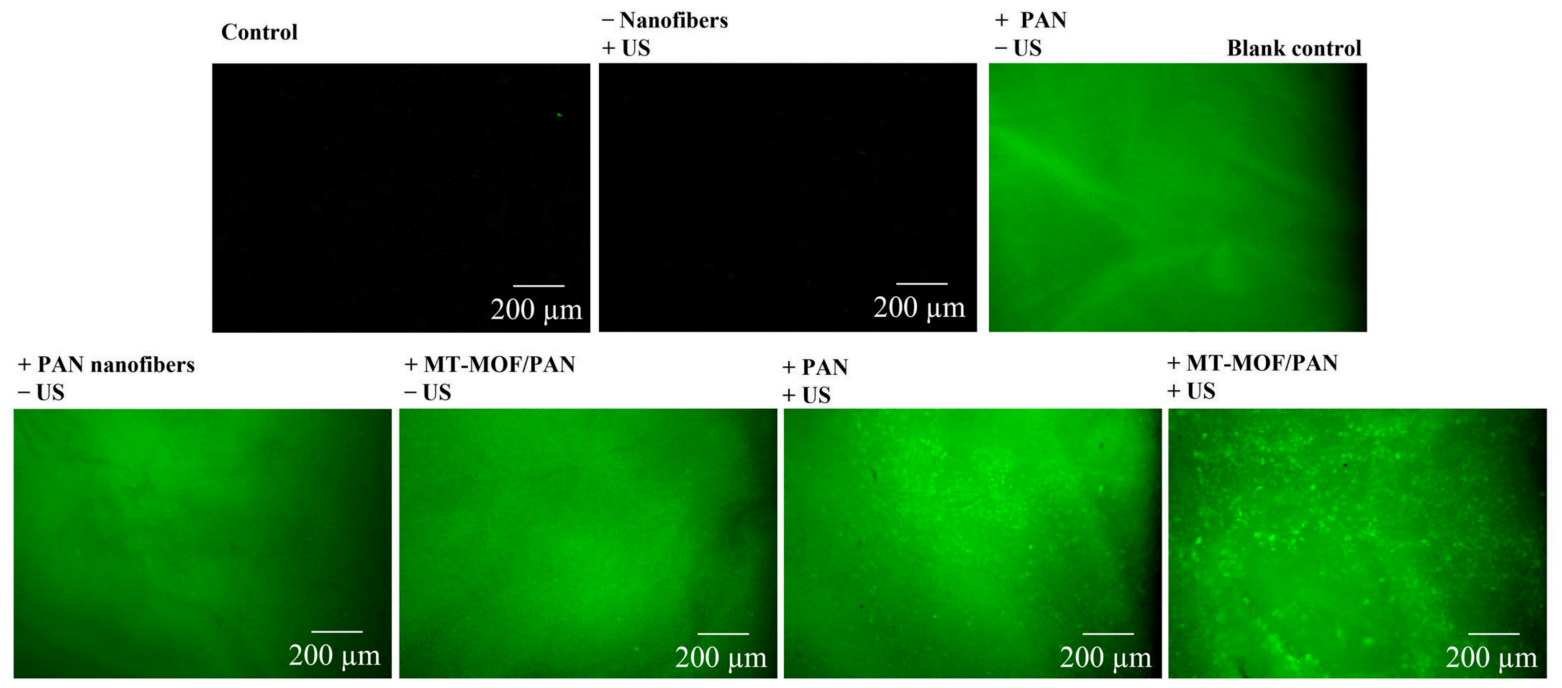
Detection of Ca^2+^ influx in macrophages in the control, US, PAN, MT-MOF/PAN, PAN + US, MT-MOF/PAN + US groups.

The enhanced secretion of TNF-α and IL-6 in the MT-MOF/PAN + US group was consistent with established evidence that electrical stimulation activates the NF-κB signaling pathway in macrophages. Mechanistically, electrical stimulation promotes Ca^2+^ influx, which regulates transcriptional programs, including NF-κB activation and inflammatory cytokine production [36]. The piezoelectric effect was hypothesized to specifically activate ion channels and increase intracellular Ca^2+^ concentration. To verify this, Ca^2+^ influx in RAW-Blue cells grown on PAN nanofibers and MT-MOF/PAN nanofibers was detected by Fluo-4 AM staining after 40 min of US treatment (Figure 6). The nanofibers themselves absorbed the Fluo-4 AM probe, resulting in a high background brightness. Consequently, absolute fluorescence intensity in this assay could not be reliably used for precise quantitative comparison across groups. An additional blank group containing nanofibers but without cell seeding was included as a reference. Against this background, both the PAN and MT-MOF/PAN groups showed numerous distinct fluorescent cells only under US stimulation. The MT-MOF/PAN group exhibited a stronger signal than the PAN group, indicating that the combination of nanofibers and US, rather than either alone, was required to trigger this intracellular response. The electric stimulation generated by MT-MOF/PAN nanofibers under US excitation activated ion channels, leading to a transient elevation of intracellular Ca^2+^, which in turn triggered NF-κB mediated inflammatory signaling and drove the intended M1 pro-inflammatory polarization. This response, reflected by the upregulated secretion of TNF-α and IL-6, underlies the antitumor immunomodulatory strategy of this work. Collectively, these findings revealed a mechanistic link among US-driven piezoelectric stimulation, intracellular Ca^2+^ influx, and pro-inflammatory macrophage activation on MT-MOF/PAN nanofibers, although this remains to be confirmed by inhibitor studies.

## 4. Conclusions

In summary, we developed MT-MOF/PAN composite nanofibers that function as wireless piezoelectric transducers, converting US energy into localized electrical signals that promote macrophage M1 polarization. Upon US excitation, the MT-MOF/PAN nanofibers activated the NF-κB pathway and stimulated the secretion of TNF-α and IL-6 relative to controls, without compromising cell viability. Mechanistic investigation suggested that piezoelectrically induced Ca^2+^ influx and subsequent NF-κB activation underlie the observed inflammatory response. These findings provide proof-of-concept in vitro evidence that MT-MOF/PAN nanofibers constitute a viable wireless and electrode-free antitumor immunomodulatory platform. As the proposed mechanism is based on protein-expression-level evidence, systematic M1/M2 phenotypic marker profiling is required to validate these findings before establishing their translational potential.

## Data Availability

The original contributions presented in this study are included in the article/supplementary material. Further inquiries can be directed to the corresponding author.

## Supplementary Materials

The following supporting information can be downloaded at https://www.mdpi.com/article/10.3390/nano16140853/s1: Figure S1. (a) Scanning electron microscopy (SEM) of MT-MOF, (b) Dynamic light scattering (DLS) of MT-MOF. Figure S2. (a) Diameter distribution of PAN nanofibers, (b) Diameter distribution of MT-MOF/PAN nanofibers. The nanofibers diameters were analyzed using ImageJ software (n = 50 per group). Figure S3. Quantitative statistical analysis of (a) spread area, (b) Solidity of cells cultured on TCP, PAN, and MT-MOF/PAN nanofibers (n = 30 randomly selected cells per group). Figure S4. Representative fluorescence images of intracellular ROS levels detected by ROS kit in cells subjected to different treatments. Green fluorescence intensity indicates the relative ROS level. Groups: Control, − Nanofibers + US; + H_2_O_2_ − US (oxidative stress model), + H_2_O_2_ + PAN, + PAN − US, + MT-MOF/PAN − US, + PAN + US, + MT-MOF/PAN + US. Scale bar = 200 μm. Figure S5. Hemolysis assay of PAN and MT-MOF/PAN nanofibers. (a) Representative photographs of red blood cells (RBCs) after incubation with PBS (negative control), Triton X-100 (positive control), PAN, and MT-MOF/PAN, followed by centrifugation. (b) Quantitative hemolysis ratios (%) of RBCs treated with PBS, PAN, MT-MOF/PAN, water, and Triton X-100 (n = 3).

## Abbreviations

The following abbreviations are used in this manuscript:

MT-MOF	Manganese–titanium metal–organic frameworks
PAN	Polyacrylonitrile
US	Ultrasound
NF-κB	Nuclear factor-κB
IL-6	Interleukin-6
TNF-α	Tumor necrosis factor-alpha
MOF	Metal–organic frameworks
SEM	Scanning electron microscopy
BSE-TP	Backscattered electron scanning electron microscopy images of two-dimensional planar membrane
EDS	Energy-dispersive X-ray spectroscopy
FTIR	Fourier transform infrared spectroscopy
XRD	X-ray diffraction
ELISA	Enzyme-linked immunosorbent assay
