# Supplementary material for: Ultrasound-Activated Metal–Organic Frameworks Incorporated Polyacrylonitrile Nanofibers Promote Macrophage Inflammation
Source: Nanomaterials (Basel). 2026 Jul 11;16(14):853. doi: 10.3390/nano16140853 (PMC13415845; doi:10.3390/nano16140853)
Supplement: Supplementary file 1 [file nanomaterials-16-00853-s001.zip › nanomaterials-4390773-supplementary.pdf]

nanomaterials

MDPI

Supporting Information

Ultrasound-Activated Metal-Organic Frameworks Incorporated Polyacrylonitrile Nanofibers Promote Macrophage Inflammation

Shiqin Dai [1,2], Nao Kawata [1,2], Ahmed Nabil [1] and Mitsuhiro Ebara [1,2,3,4,*]

1 Research Center for Macromolecules and Biomaterials, National Institute for Materials Science (NIMS), Tsukuba, Ibaraki 305-0044, Japan
2 Graduate School of Pure and Applied Sciences, University of Tsukuba, Tsukuba, Ibaraki 305-0006, Japan
3 Department of Materials Science and Technology, Tokyo University of Science, Tokyo 125-8585, Japan
4 Department of Biochemistry and Microbiology, Faculty of Pharmaceutical Sciences, Chulalongkorn University, Bangkok 10330, Thailand
* Correspondence: Ebara.Mitsuhiro@nims.go.jp;

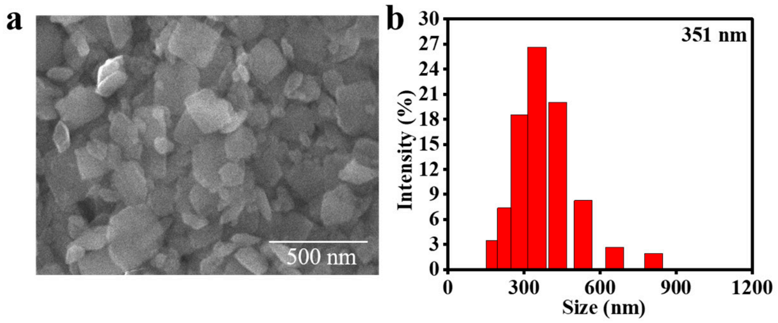

Figure S1. (a) Scanning electron microscopy (SEM) of MT-MOF, (b) Dynamic light scattering (DLS) of MT-MOF.

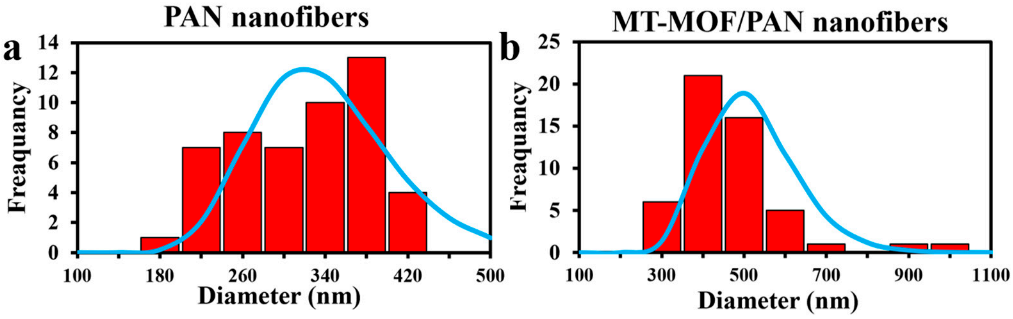

Figure S2. (a) Diameter distribution of PAN nanofibers, (b) Diameter distribution of MT-MOF/PAN nanofibers. The nanofibers diameters were analyzed using ImageJ software (n = 50 per group).

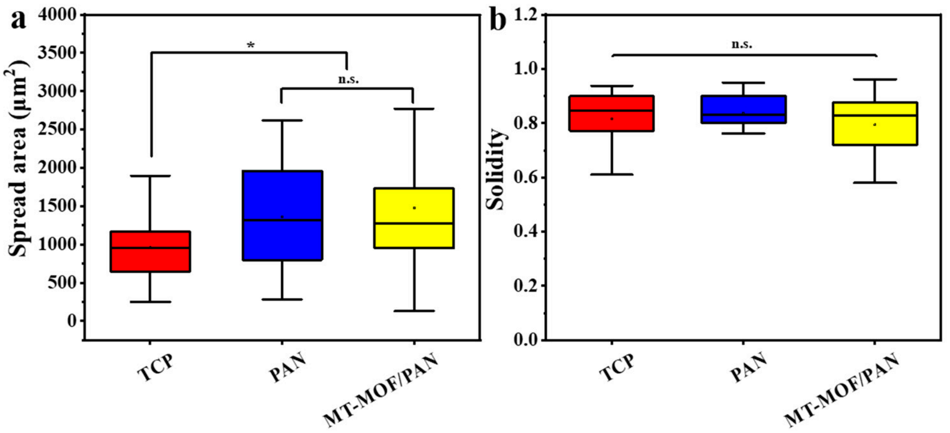

Figure S3. Quantitative statistical analysis of (a) Spread area, (b) Solidity of cells cultured on TCP, PAN, and MT-MOF/PAN nanofibers (n = 30 randomly selected cells per group).

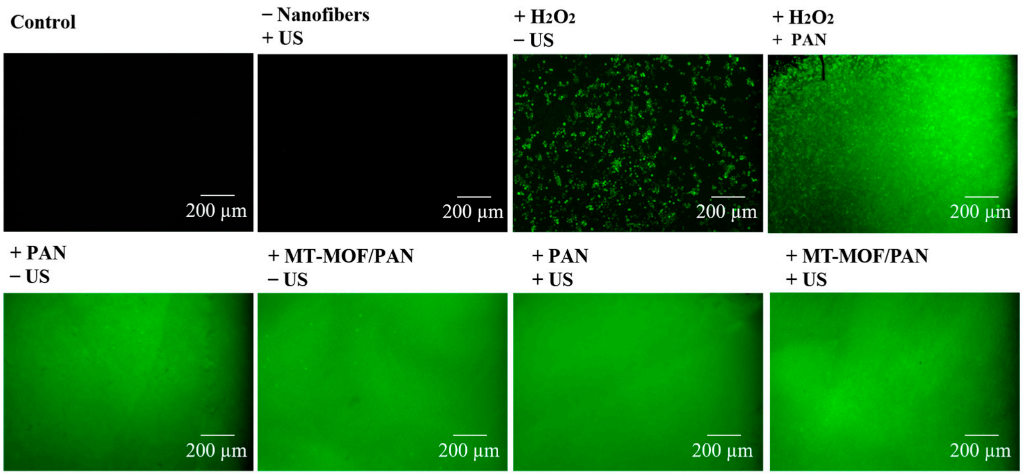

Figure S4. Representative fluorescence images of intracellular ROS levels detected by ROS kit in cells subjected to different treatments. Green fluorescence intensity indicates the relative ROS level. Groups: Control, − Nanofibers + US; + H_2O_2 − US (oxidative stress model), + H_2O_2 + PAN – US, + PAN – US, + MT-MOF/PAN – US, + PAN + US, + MT-MOF/PAN + US. Scale bar = 200 μm.

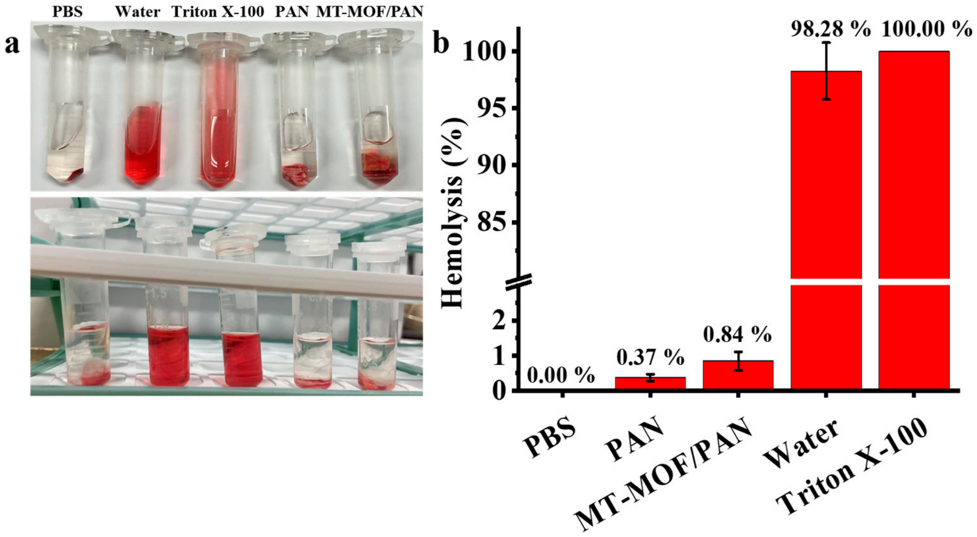

Figure S5. Hemolysis assay of PAN and MT-MOF/PAN nanofibers. (a) Representative photographs of red blood cells (RBCs) after incubation with PBS (negative control), water, Triton X-100 (positive control), PAN, and MT-MOF/PAN, followed by centrifugation. (b) Quantitative hemolysis ratios (%) of RBCs treated with PBS, water, Triton X-100, PAN, and MT-MOF/PAN (n = 3).
